# Detection of Gold Nanoparticles Aggregation Growth Induced by Nucleic Acid through Laser Scanning Confocal Microscopy

**DOI:** 10.3390/s16020258

**Published:** 2016-02-19

**Authors:** Ramla Gary, Giovani Carbone, Gia Petriashvili, Maria Penelope De Santo, Riccardo Barberi

**Affiliations:** 1 Physics Department, University of Calabria, Rende 87036, Italy; giovanni.carbone@gmail.com (G.C.); g.petriashvili@yahoo.co.uk (G.P.); maria.desanto@fis.unical.it (M.P.D.S.); riccardo.barberi@fis.unical.it (R.B.); 2 Institute of Cybernetics of the Georgian Technical University, Euli str. 5, 0175 Tbilisi, Georgia; 3 CNR-Nanotec UOS di Cosenza, c/o University of Calabria, Rende 87036, Italy

**Keywords:** gold nanoparticle aggregation, deoxyribonucleic acid, laser scanning confocal microscopy, surface plasmon resonance, Förster resonance energy transfer, hydrophobicity

## Abstract

The gold nanoparticle (GNP) aggregation growth induced by deoxyribonucleic acid (DNA) is studied by laser scanning confocal and environmental scanning electron microscopies. As in the investigated case the direct light scattering analysis is not suitable, we observe the behavior of the fluorescence produced by a dye and we detect the aggregation by the shift and the broadening of the fluorescence peak. Results of laser scanning confocal microscopy images and the fluorescence emission spectra from lambda scan mode suggest, in fact, that the intruding of the hydrophobic moiety of the probe within the cationic surfactants bilayer film coating GNPs results in a Förster resonance energy transfer. The environmental scanning electron microscopy images show that DNA molecules act as template to assemble GNPs into three-dimensional structures which are reminiscent of the DNA helix. This study is useful to design better nanobiotechnological devices using GNPs and DNA.

## 1. Introduction

Individual particles and assemblies of gold nanoparticles (GNPs) show different optical and electrical properties. For instance, the color change due to aggregation of GNPs is an indicator of the surface plasmon shift, allowing potential applications in sensors to recognize DNA or its components [1].

Life processes strongly depend on nucleic acids and proteins which, therefore, are used as probes for measurements of other components in biological systems [2]. The development of nanobiotechnology is helped by a deep understanding of the interaction of GNPs with nucleic acids, which are used as a basic tool for nanobiotechnological devices [3,4]. Examples of this approach are the organization of metal and semiconductor nanoclusters [5], several bioanalytical techniques [6], the biomolecular electronics [7] and nanomechanical systems. Therefore, the DNA efficiency for inducing GNPs self-assembly is a very interesting subject of research. J. Yang *et al*. [8] demonstrated how single-stranded DNA could be used to manage the placement of two different sized GNPs in a complex particle system. K. N. Ganesh *et al*. [9] have studied electrostatic assembly of cationic surfactants-capped GNPs on DNA duplex. H. Nakao *et al.* [10] have reported the highly-ordered assemblies of GNPs in well-aligned and long-range order on DNA molecules.

Color change, UV-VIS spectroscopy, and dynamic light scattering have been used as techniques for the detection of GNPs aggregation [11,12,13]. Confocal and two photon microscopies and single-cell plasmonically-enhanced Rayleigh-scattering imaging spectroscopy were used as imaging techniques to observe the GNPs aggregation after their accumulation within living cells through endocytosic processes [14,15,16]. These approaches take advantage of the characteristic optical properties of plasmonic GNPs, namely, their ability to strongly scatter light, and the coupling of their plasmonic fields when particles come into close proximity.

In this work, the laser scanning confocal microscopy (LSCM) is used to observe the double-stranded DNA induced aggregate growth of cationic surfactants-capped GNPs. The observation based on the light scattering enhancement from GNPs aggregates is not recommended in our experiments since there is a strong overlap between the light scattering from DNA/cationic surfactants complexes and the light scattering from GNPs aggregates (see in Appendix). Therefore, to skirt this overlap problem we detected the GNP aggregation through the Förster Resonance Energy Transfer (FRET) process. Nile Blue perchlorate (NB) is used as a probe because, in addition to the NB molecules ability to be intercalated inside the DNA helix [17,18], it seems that this probe can be also entrapped within the hydrophobic part of the surfactants bilayer films coating the aggregated GNPs. This entrapment places this probe in proximity to metallic surfaces leading to FRET. Due to the strong interaction between NB molecules and the large localized fields induced by the plasmonic coupling, a highly enhanced fluorescence is produced allowing the localization of GNPs aggregates. 

## 2. Preparation of Samples and Methods

### 2.1. Gold Nanoparticles 

Our experiments are performed by using commercially available GNPs, obtained from Sigma Aldrich as stabilized suspension in citrate buffer with a core size of 37–43 nm and a mean hydrodynamic diameter of 48–56 nm. GNPs are usually prepared by using citrate reduction of Au ions in an aqueous solution; therefore, GNPs surface is negatively charged. To minimize their aggregation, the versatile surface chemistry of citrate-capped GNPs allows them to be coated with a hydrophilic bilayer film on their surface via electrostatic interaction. By using a stabilizer, such as cetyltrimethylammonium bromide (CTAB), which presents a positive hydrophilic ammonium head group and a hydrophobic long hydrocarbon tail, the surface electric charge is converted from negative to positive [19,20]. 

**Figure 1:**
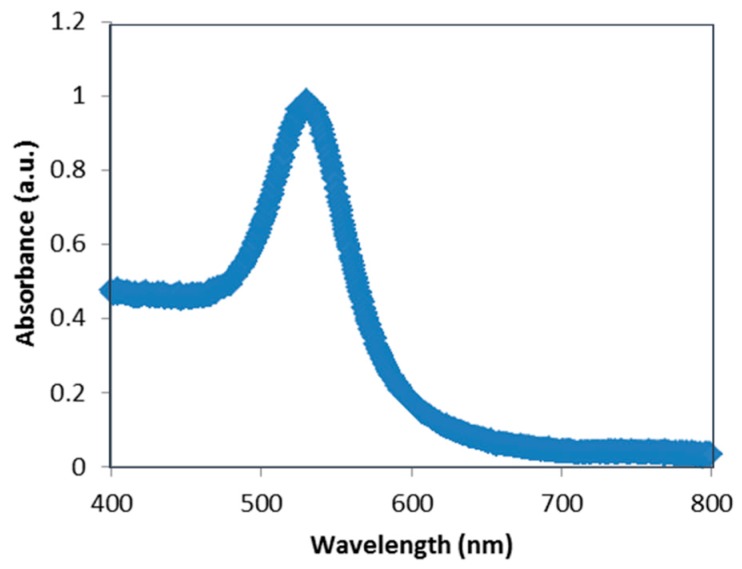
Absorption spectrum of GNPs stabilized suspension in citrate buffer.

The absorption spectrum of the solution containing the dispersed GNPs (Figure 1) was observed through a UV-VIS-NIR spectrophotometer AVASPE-2048 Avantes. Surface plasmon resonance (SPR) causes strong scattering as well as absorption of light. Therefore, the absorption spectrum of dispersed GNPs provides data about the SPR band of GNPs. Our observations show that SPR band of the GNPs used for this work ranges from 400 nm to 600 nm, with a peak at 525 nm (Figure 1).

### 2.2. Preparation of the DNA Solution

Nucleic acid solution was prepared by dissolving 10 mg of commercially available deoxyribonucleic acid (double-stranded (ds) molecule), DNA, fish sperm from salmon testes (Sigma-Aldrich) in 10 mL of doubly distilled water. 24 h or more were needed for the dissolution at 4 °C, accompanied by occasional gentle shaking.

### 2.3. LSCM Measurements

**Figure 2:**
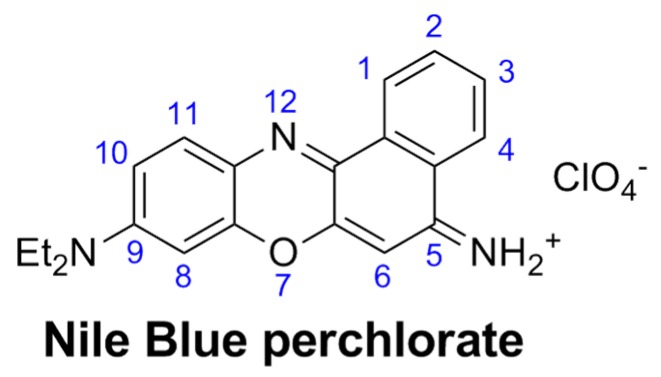
Molecular structure of the fluorescence probe NB.

For the observation of the GNPs aggregation growth induced by DNA based on the NB fluorescence analysis, four solutions were prepared. The DNA/GNPs/NB solution is prepared at first. We added to 1.6 g of the GNPs stabilized suspension in citrate buffer, 0.002 g of the crystallized probe Nile Blue A perchlorate (NB; Figure 2) purchased from Sigma-Aldrich. This mixture is left in a shaker for 15 min at 50 °C in order to dissolve the probe. Then, 1.3 mL from the dissolved DNA was transported to the previous GNPs/NB mixture. The three other solutions were prepared for control experiments. The first one was prepared with the same procedure described above except that this mixture was free from DNA. To have the same concentration of GNPs in DNA/GNPs/NB solution described previously, 1.3 mL of double distilled water was added to GNPs/NB solution (solution No. 1 for control experiments).

To study the effect of GNPs on the fluorescence of NB bound to the cationic surfactants present as stabilizer in the GNPs suspension in citrate buffer, the solution No. 2 was prepared with the same procedure described above for the GNPs/NB solution (solution No. 1) except that GNPs were removed from the buffer solution by centrifugation at 3000 rpm for an hour. The final solution for control experiments (solution No. 3) was prepared by adding 0.002 g of NB to 1.3 mL from the dissolved DNA. Then, the resulting solutions were gently shaken at room temperature for 5 min, stored at 4 °C for 48 h, and 0.25 mL from each solution was deposited by drop-coating to the glass slide treated by deionized water. The four coated films on substrate were stored for 24 h at room temperature to let the water evaporate completely resulting to four precipitates.

(LSCM) was used to image the fluorescent emission on each of the four samples. All samples were imaged on a Leica TCS SP8 using a 25 × /0.95 numerical aperture water immersion objective and identical setting. The small droplet of the immersive water was placed on the opposite side of the coated film on the glass slide with the thickness of 1.13 mm. The 514 nm was used as pump beam and the fluorescent emission was sent simultaneously to 2 PMTs (PMT1 and PMT2) collecting respectively the light in the (550–600) nm and (600–750) nm ranges. The SPR of GNPs and the excitation spectrum of NB have respectively the (400–600) nm and (470–690) nm ranges [21]. Thus, 514 nm was chosen for the irradiation because this wavelength is within these two ranges.

Since the NB emission depends on the polarity of the environment which is red shifted with higher polarity, emitted light was detected in (550–600) nm and (600–750) nm ranges [17]. The use of two PMTs allowed the simultaneous acquisition of two images. In order to enhance the contrast, each image is colorized by an artificial color, according to the detection ranges of PMT1 and PMT2, where the green or the yellow areas are the expression of the fluorescence from NB bound to the hydrophobic part of cationic surfactants and the red or the grey domains are the expressions of NB bound to DNA.

### 2.4. Scanning Electron Microscopy 

An environmental scanning electron microscope (ESEM) Quanta 400F installed at Nanotec-Cosenza (Italy) by FEI Europe B.V ( Achtseweg Noord, 5, 5651 GG Eindhoven, The Netherlands) and FEI Italy (Srl Viale Bianca Maria, 23, 20122 Milano (Mi)) Companies (Milano, Italy) has been used to image the samples prepared with GNPs at pressure of 1 mbar and voltage 5–10 kV. 

## 3. Experimental Results and Discussion

### 3.1. Images of LSCM and Fluorescence Spectroscopy 

#### 3.1.1. Effect of Gold Nanoparticles on the Fluorescence of NB Bound to Cationic Surfactants

**Figure 3:**
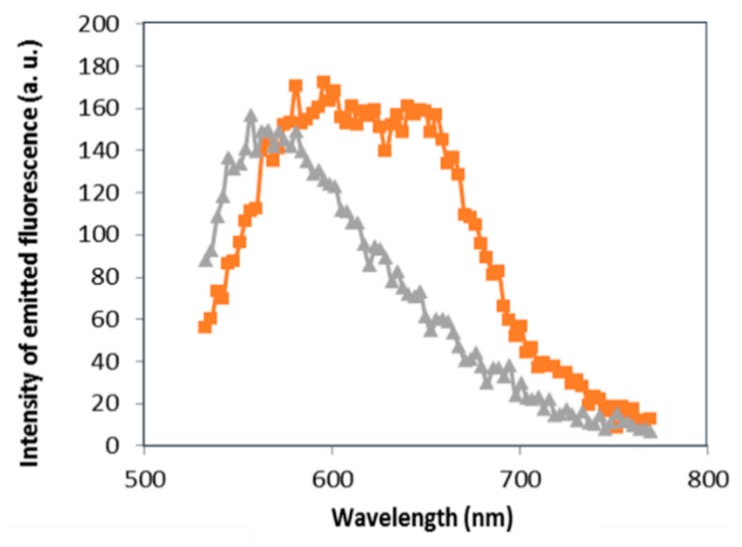
Effect of gold nanoparticles on the fluorescence spectra of NB bound to cationic surfactants complexes (grey and orange curves are recorded respectively from precipitations of aggregated cationic surfactants complexes without and with GNPs). Emission obtained from 514 nm excitation (lambda scan mode from a confocal microscope) and detected in the (530–775) nm range.

The evaporation of the coated films prepared from the solutions No. 1 and 2 induced, respectively, a precipitation of aggregated cationic surfactants complexes with and without GNPs. Before the drying of the coated film (solution No. 2), NB as hydrophobic probe moves closely to the hydrophobic part of cationic surfactants [17]. As depicted in Figure 3, the fluorescence spectra of the untreated GNPs precipitation recorded with the lambda scan mode across the (530–775) nm range under an excitation of 514 nm shows a maximum at the wavelength of 565.59 nm (grey curve, Figure 3). Under the same excitation, the fluorescence spectra of treated GNPs precipitation recorded with the lambda scan mode across the same range is broadened and slightly redshifted compared to the fluorescence spectra in the absence of GNPs (orange curve, Figure 3). For the sample treated with GNPs, the evaporation of the solution induced their aggregation which was expected since that the dipole-dipole interaction between these nanoparticles is stronger when the distance between them decreases. The SPR of GNPs presents a broadening and red shift when they aggregate [22]. Moreover, it seems that NB entrapment inside the hydrophobic part of the bilayer polymeric film covering aggregated GNPs is responsible of a FRET between NB (acceptor) and aggregated GNPs (donor) which explains the fluorescence enhancement in the (565.59–706.59) nm range (Figure 3). When a fluorophore is quite close to a plasmonic particle, for instance within 10 nm, the electrons of the fluorophore are influenced by the plasmon field changing their excitation/emission properties. The interaction results in a quenching or enhancement of the fluorescence [23,24], which improves the performance of the optical response of the fluorescent contrast agents [25,26,27].

Thus, the FRET process between aggregated GNPs and NB induced a broadening of the fluorescence spectra of NB bound to cationic surfactants complexes.

Moreover, the slightly redshift of the fluorescence spectra of NB bound to cationic surfactant complexes in the presence of GNPs can be attributed to the SPR redshift of GNPs after their aggregation. 

#### 3.1.2. Effect of DNA on GNPs Aggregation

In the previous paragraph we showed that the broadening and the redshift of the fluorescence spectra of NB bound to cationic surfactants complexes was an indicator of GNPs aggregation. 

In the following paragraph, the effect of DNA on GNPs aggregation size will be discussed. 

**Figure 4:**
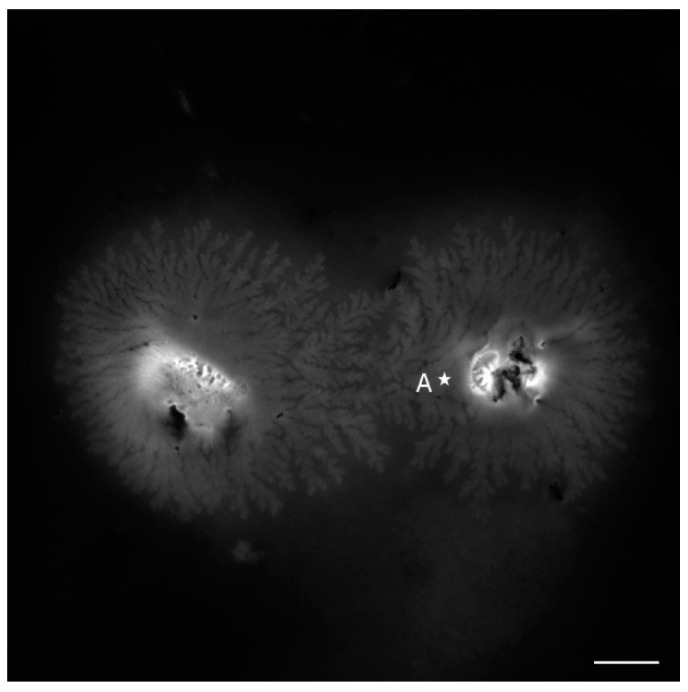
LSCM image of precipitated DNA (evaporated solution No. 3). The 514 nm was used as the pump beam and the fluorescent emission was detected in the (550–650) nm and (650–750) nm ranges. The scale bar donates 40 µm.

**Figure 5:**
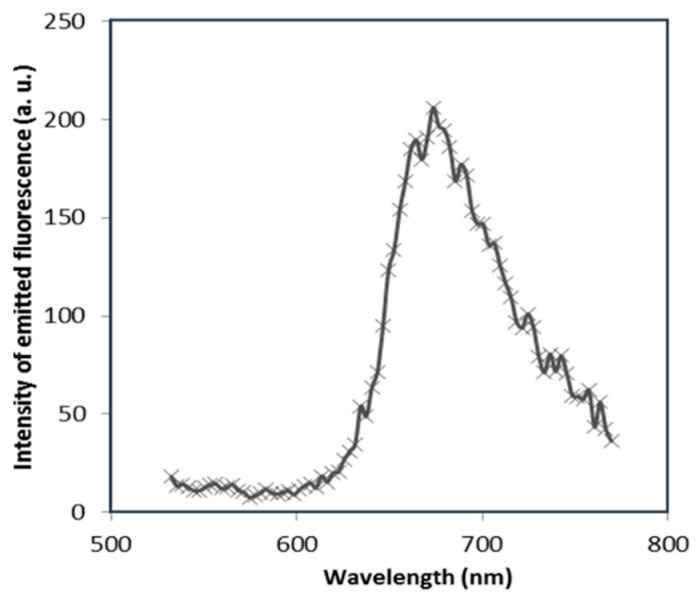
Fluorescence spectra of NB bound to DNA molecules organized into liquid crystalline phase recorded with lambda scan mode from the point marked as A (Figure 4). Emission obtained from 514 nm.

As the DNA is concentrated (>1 mg/mL; solution No. 3), the molecules were precipitated into a liquid crystalline phase after the drying of the solution deposited via drop-coating on the glass slide [28]. Figure 4 shows emitted fluorescence from NB bound to DNA molecules organized into the flower like texture which is a highly ordered liquid crystalline form of DNA [29], detected in the (550–750) nm range under the wavelength of 514 nm. As depicted in Figure 5, the fluorescence spectra of NB bound to DNA molecules organized into liquid crystalline phase recorded with lambda scan mode from point marked as A is redshifted compared to the fluorescence spectral of NB bound to cationic surfactants complexes (Figure 3). This shift is explained by the sensitivity of NB to the polarity of the environment. The hydrophobic part of cationic surfactants, where NB is placed is non-polar, whereas in the case of DNA stained with NB, there is an amount of this probe which is bound to the polar negatively-charged phosphate backbone.

**Figure 6:**
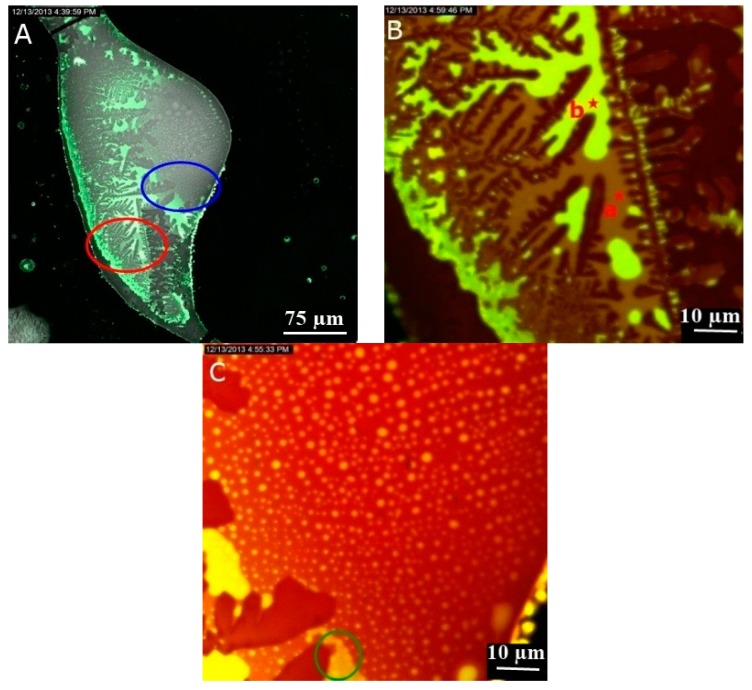
LSCM images of precipitated DNA/GNPs/solution. (**B**) and (**C**) are images of higher magnification at respectively the red and blue markets location in image (**A**). The 514 nm was used as pump beam. Light detected in (550–650) nm is green (Figure 6A,B) or yellow (Figure 6C) and light detected in (650–750) nm is red (Figure 6B,C) or grey (Figure 6A). The representation colors have been chosen to enhance the contrast.

Figure 6 shows the emitted fluorescence, detected in the (550–750) nm ranges, from precipitated dried DNA/GNPs/NB solution under the excitation wavelength of 514 nm. A strong emitted light enhancement (green and yellow regions in Figure 6) is detected in the (550–560) nm range compared to the untreated sample with GNPs (Figure 4 and Figure 5). The emitted light detected in this range is attributed to an amount of NB entrapped inside the hydrophobic part of cationic surfactants stabilizing the GNPs suspension in citrate buffer. Moreover, as shown inside the region marked with the green color in Figure 6C, brighter spots are detected which suggested that GNPs are aggregated into spherical clusters. When NB are placed at proximity to the GNPs surface, a fluorescence enhancement is induced due to the FRET process. Additionally, GNPs plasmonic fields couple together when they are in close proximity [16]. For denser GNPs clusters, the plasmonic field coupling is increased, resulting in significantly higher absorption [30], which induced a higher FRET and brighter signal. 

**Figure 7:**
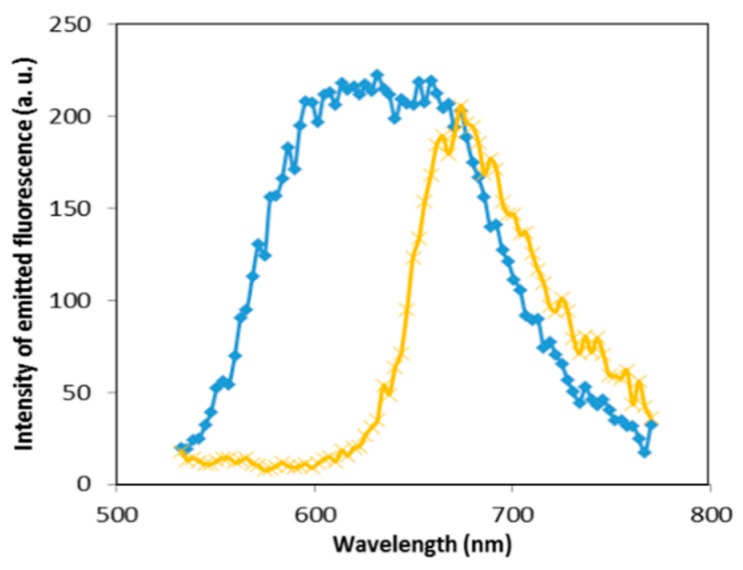
Fluorescence spectra of NB bound to precipitated DNA in the absence (yellow) and in the presence (blue) of GNPs aggregation recorded with lambda scan mode in respectively points marked as a and b in Figure 6B. The emission is obtained under an excitation of 514 nm and it is detected in the (530–775) nm range.

As shown in Figure 7, the fluorescence spectra recorded at the point marked as in Figure 6B is the same in point marked as A in Figure 4, whereas the fluorescence spectra recorded at the point marked as b in Figure 6B is broadened and blueshifted (Figure 7). As explained in the previous paragraph, the broadening of the fluorescence spectra is an indicator of GNPs aggregation. When GNPs are aggregated in the presence of DNA, an amount of NB inside the hydrophobic part of cationic surfactant bilayer film surrounding the GNPs is responsible of the blueshift.

The fluorescence spectra recorded from aggregated GNPs in the presence of DNA (Figure 7 blue curve) is slightly redshifted compared to the fluorescence spectra recorded from aggregated GNPs without DNA (Figure 3 orange curve).

This redshift seems to be attributed to a growth of GNPs aggregation size induced by DNA compared to the spontaneous smaller GNPs aggregation induced by dipole-dipole interaction.

### 3.2. Images of ESEM

**Figure 8:**
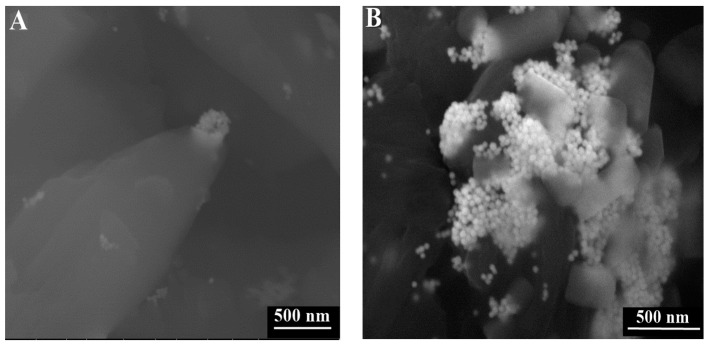

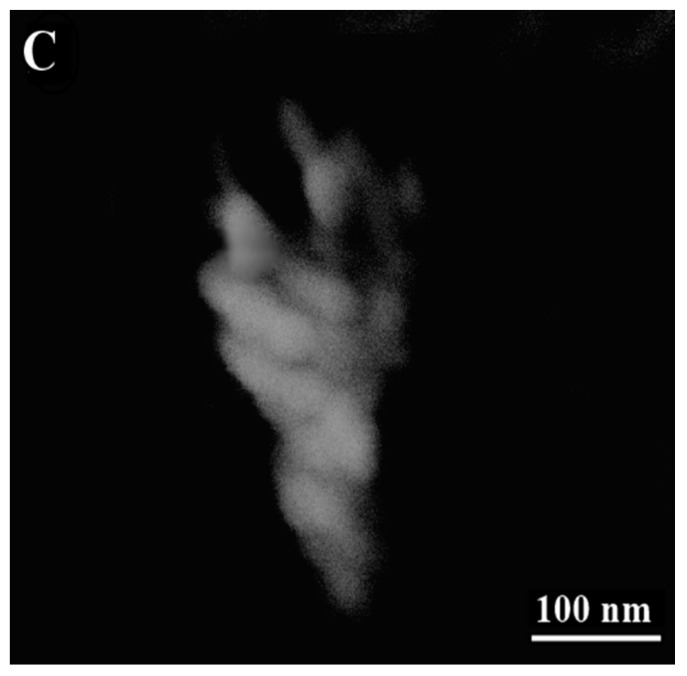
ESEM images of precipitates of evaporated; (**A**) GNPs-stabilized suspension in citrate buffer; (**B**,**C**) DNA/GNPs solutions.

After their observations with confocal microscopy, the two precipitations of GNPs stabilized suspension in citrate buffer with and without DNA were stored at 4 °C for 24 h before ESEM observations. Figure 8A shows that for the sample untreated with DNA, GNPs are aggregated into some clusters. As previously discussed, this self-assembly is due to a dipole-dipole interaction between nanoparticles. In the presence of DNA, the GNPs aggregation size increased. GNPs are aggregated into denser three-dimensional clusters (Figure 8B). Moreover, for a higher magnification (100 nm scale bar), GNPs are assembled in 3-D structures following the helix morphology of DNA (Figure 8C). This aggregation growth is due to electrostatic and hydrophobic interactions between positive charged hydrophobic head groups of the bilayer cationic surfactants film around GNPs and negatively-charged DNA phosphate backbone [9,31].

## 4. Conclusions

In this paper, FRET between GNPs (donors) and dyes (acceptors) is used for the detection of GNPs aggregation through LSCM. Moreover, DNA form Salmon tests was tested for the GNPs aggregation growth. The 514 nm was used as pump beam to irradiate the sample, since this wavelength is within the SPR of GNPs and the excitation bandwidth of the fluorescence dye (NB). 

Thus, the fluorescence was manipulated with the SPR through the FRET process. The fluorescence spectra and peak are, respectively, broadened and shifted, which allowed the detection of the GNPs aggregation. In the presence of DNA, the fluorescence spectrum is more redshifted due to the size increase of GNPs aggregation induced by DNA. This result is confirmed with ESEM images which also showed a GNPs assembly into denser clusters in the presence of DNA. Moreover, for a higher ESEM image magnification, GNPs are assembled in 3-D structures following the helical morphology of DNA. DNA induced GNPs aggregation growth due to the electrostatic and hydrophobic interactions between positively-charged head groups of cationic surfactants coating GNPs and negatively-charged DNA phosphate backbone. Furthermore, these results may be helpful in the world of nanobiotechnology (gene delivery, cancer therapy, etc) mainly that the DNA-binding NB molecules are not toxic [17,18] and aggregated GNPs were usually detected via light scattering techniques, which can present problems since several structures within biological tissues also scatter light strongly. Thus, FRET between GNPs and dyes detected through LSCM result to be ideal for further bio-applications to discriminate aggregated GNPs and complex structures of tissue and cells.

## Figures and Tables

**Figure A1:**
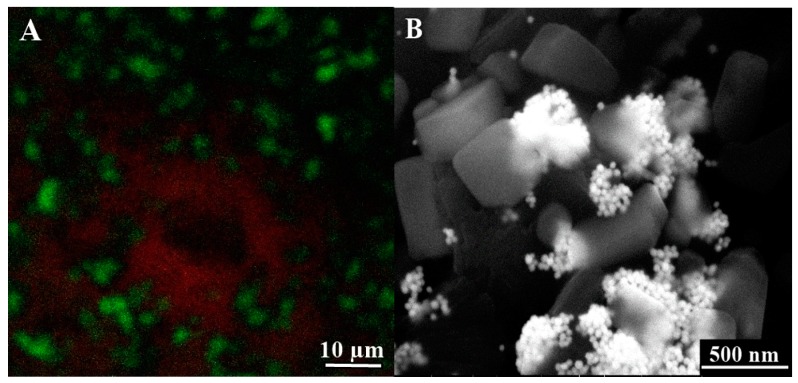
The LSCM and ESEM images of the evaporated DNA/GNPs solution precipitate; (**A**) LSCM image. The 514 nm was used as the pump beam. Green spots correspond to scattered light from CTAB/DNA complexes and GNPs aggregations. The red area corresponds to emitted light from NB bound to DNA molecules, organized in a liquid crystalline phase, detected in the 650–750 nm range; and (**B**) ESEM image. The DNA/CTAB complexes are in the form of cubic structures.

## Appendix

The excess of the CTAB amount introduced as a stabilizer in the citrate buffer of the GNPs suspension is responsible for a strong interaction of these surfactants molecules with DNA which induced CTAB/DNA complexes in the form of cubic structures as depicted in Figure A1B. In addition to the strong light scattering from aggregated GNPs, the DNA/CTAB complexes also scatter light. This strong overlap makes impossible the detection of aggregated GNPs with the light scattering technique in the presence of CTAB and DNA compounds (Figure A1A). Therefore, to skirt this overlap problem, we detected the GNPs aggregation through the FRET process, observing the resulting enhanced fluorescence produced by GNPs aggregates.
